# Surgical provider-reported reasons for utilization of the World Health Organization’s Surgical Safety Checklist at a tertiary hospital in Ghana

**DOI:** 10.1371/journal.pgph.0001143

**Published:** 2023-01-12

**Authors:** Eyram Cyril Bansah, Kekeli Kodjo Adanu, David Adedia, Adolphina Addoley Addo-Lartey

**Affiliations:** 1 Department of Surgery, Richard Novati Catholic Hospital, Sogakope, Ghana; 2 Department of Surgery, School of Medicine, University of Health and Allied Sciences, Ho, Ghana; 3 Department of Basic Sciences, School of Basic and Biomedical Sciences, University of Health and Allied Sciences, Ho, Ghana; 4 Department of Epidemiology and Disease Control, University of Ghana School of Public Health, Accra, Ghana; University of California San Francisco, UNITED STATES

## Abstract

Despite the established positive benefits, LMICs’ adoption of the WHO Surgical Safety Checklist (SSC) is inadequate, with as little as 20% use. This study assessed the utilization and beliefs that drive the non-utilization of the WHO SSC among surgical providers at Korle Bu Teaching Hospital (KBTH) in Accra, Ghana. A cross-sectional study was conducted among 186 surgical providers at the KBTH in Ghana. Data collected included the category of personnel, awareness of the SSC, training received, previously identified barriers, and staff perceptions. Utilization and drivers associated with non-utilization of the SSC were assessed using bivariate log-binomial regression. Out of 190 surgical professionals invited, 186 gave their consent and participated in the survey, giving a response rate of 97.9%. Respondents comprised 69 (37%) surgeons, 66 (36%) anesthetists, and 51 (27%) nurses. Only 30.4% of surgical professionals always use the SSC, as advised by WHO. The majority (67.7%) of surgical professionals had received no formal training on using the WHO SSC. The proportion was highest among surgeons (81.2%) compared to anesthetists (66.7%) and nurses (51%). Surgeons were perceived by other professionals to be the least supportive of checklist use (87.6%), in contrast to nurses (96.1%) and anesthetists (93.9%). Significant drivers associated with checklist usage among surgical professionals included the SSC taking too long to complete, poor communication between anesthetist and surgeon, checklist not covering all perioperative risks, difficulty finding a coordinator, poor attitude of team members toward questions, surgical specialty/unit and training status of professionals. The checklist was always used by only a small (30%) proportion of surgical professionals at the KBTH. Improving checklist use will necessitate its careful application to all surgical operations and a cycle of periodic training that includes context-specific adjustments, checklist auditing, and feedback from local coordinators.

## Introduction

Surgical complications are a major source of disability and death, placing a significant economic burden on patients and service providers [1]. By one estimate, more than 266 million surgeries are conducted worldwide every year [2]. Recent studies by the International Surgical Outcome Study and the African Surgical Outcome Study suggest a surgical complication rate of 16.8% and 18.2%, respectively [3, 4]. As part of its efforts to reduce postoperative adverse events and increase patient safety, the WHO’s World Alliance for Patient Safety, through its Safe Surgery Saves Lives campaign introduced the Surgical Safety Checklist (SSC) in 2008 [5]. The purpose of the checklist is to improve patient safety by ensuring some basic minimum surgical standards that prevent avoidable surgery-related complications [6]. As a standardization tool for operating room safety protocols, checklists are acknowledged to be straightforward, plain, and easy to reproduce [7]. In addition, checklists are a cost-effective innovation for improving surgical patients’ safety and outcomes that can be used across different economic settings [8].

Following its universal deployment, the WHO SSC is the most extensively utilized of the various checklists available in healthcare [9]. It is a 19-item checklist (S1 Checklist) that is used in three critical phases of a surgical procedure: sign-in–before anesthesia is administered; timeout–before the surgical incision is made; and sign-out–before the patient leaves the operating room at the end of the procedure [9, 10]. A key feature of the checklist is to bring the whole team together to share vital information throughout the surgical procedure [11]. Several actions are required of the surgical team, including verifying the patient’s name, introducing team members, confirming antibiotic prophylaxis administration, and voicing concerns about the procedure [12]. To promote its universal application, the WHO SSC is designed to encourage additions and modifications that suit local practice while ensuring that some basic standards of care are maintained [12]. The WHO SSC is now regarded as the gold standard for assessing surgical care quality and is widely used in hospitals worldwide [13].

Since its inception, the SSC’s utility and impact have been characterized by several economic circumstances and patient demographics. Data shows that it may have a more significant impact on LMICs than high-income countries [14]. Country-specific data on the impact of SSC on surgical outcomes in Sub-Saharan Africa are sparse. Nevertheless, a randomized trial found a significant reduction in maternal mortality and complication rates following the successful implementation of the checklist in South Africa [15]. Other studies undertaken following the implementation of the WHO SSC to examine the effect of implementation revealed a reduction in overall postoperative complication rates as well as certain complications such as surgical site infections, unplanned return to the operating room, and hospital fatalities [16–22]. According to the evidence so far, the WHO SSC improves patient safety by increasing better communication [23–27] among team members and minimizing medical errors in the operating room [28, 29]. However, a few studies in high-standard care settings found no significant difference in complication rates when the checklist was implemented [30, 31].

Despite its significant impact, the WHO SSC is underutilized, with compliance rates as low as 12% in some situations [32]. Unfamiliarity and fear of embarrassment, resistance from senior surgeons, hierarchy in operating rooms, checklist scheduling, and duplication of efforts have all been cited as causes for poor SSC use [33, 34]. Fourcade et al. [13] identified several barriers to checklist implementation, including the checklist being perceived as a time-consuming process with no added benefits, poor communication between team members, duplication of existing checks, uncertainty about team member roles, inappropriate nature of checklist questions for different settings, poor timing for checklist completion, increasing patient anxiety, and “gaming,” in which items are ticked off as checked even when they have not been observed. In a systematic review of the impacts and implementation of the SSC, Treadwell et al. [19] classified barriers to utilization into four categories: confusion about how to use the checklist correctly; practical changes to efficient workflow; availability of resources; and individual staff perceptions and attitudes.

There is a paucity of evidence from LMICs, where this checklist may be more valuable and practical. Melekie & Getahun [35] for example, noted a lack of training and poor coordination among surgical team members as reasons for the infrequent use of WHO SSC in an Ethiopian study. In Ghana, a quality improvement study at the Cape Coast Teaching Hospital found that, although uptake of the SSC was high (93%) about a year after its implementation, its use was complete in only 21% of all cases [36]. As a result, to boost SSC uptake and sustain its effective use in LMICs, constraints to effective utilization that are specific to the local environment must be identified. The utilization and drivers of non-utilization of the WHO SSC among surgical employees at Korle Bu Teaching Hospital (KBTH), Ghana’s largest tertiary health facility, were investigated in this study. KBTH is the foremost Teaching Hospital with a 2000-bed capacity, performing over 10,000 surgical procedures per year. It hosts the Centers of Excellence in Cardiothoracic and Plastic and Reconstructive Surgery, among other surgical specialties, and it is the main training center for over a third of the healthcare workers in Ghana, including surgeons, anesthetists, and perioperative nurses [37]. A successful implementation, therefore, may influence the acceptability and utilization of the SSC in regional and district hospitals if trained workers move and effect change in their new facilities. Identifying and removing barriers to utilization at this level may serve as a cornerstone to effectively implementing the checklist countrywide.

## Materials and methods

### Study site

In June 2019, a cross-sectional study was conducted at the Korle Bu Teaching Hospital (KBTH), Ghana’s largest tertiary referral institution. In this study, surgical providers at Korle Bu Teaching Hospital (KBTH) in Accra, Ghana, were asked about their use of the WHO SSC and the beliefs that prevent them from using it. The General Surgery, Neurosurgery, Urology and Pediatric Surgery units comprise the Surgical Department. Surgeons, anesthetists, and theatre nurses made up the surgical team. In total, there were 225 surgical employees comprising 85 surgeons, 77 anesthetists, and 63 nurses. Surgeons and anesthetists were divided into three categories based on their rank: consultant, specialist, and resident/medical officer. Consultant surgeons and anesthetists are the highest-ranked experts in their fields of endeavor appointed to take the overall responsibility for patients’ care in the hospital, while Specialists are middle-level experts who work in conjunction with consultants and resident/medical officers in the care of patients. Residents are doctors under training to become specialists, while medical officers are doctors who have completed a mandatory two-year internship (houseman ship) period after medical school and are yet to start a residency. By convention, nurses are responsible for leading the application and completion of the WHO SSC. Locally, this is usually done by the circulating nurse but may be done by any team member who is not directly involved in the surgical procedure within the sterile field. Surgeons and anesthetists have varying roles in ensuring patients’ safety, from induction of anesthesia to the completion of the surgery, including administering prophylactic antibiotics, addressing difficult airways, and minimizing blood loss. The study was open to all surgical employees in the department, provided they met the inclusion criteria.

### Inclusion and exclusion criteria

Surgical professionals were eligible to participate in the study if they had worked or studied at the facility for at least six months, were available during the study period (June 2019), and provided written informed consent. Surgical professionals refer to surgeons, anesthetists, and nurses who perform specialized roles as part of a team operating on a patient in a designated theater space. In addition, consultant surgeons and anesthetists who worked in the surgical department of the University of Ghana Medical School solely for academic purposes and had not conducted procedures in theatre for more than two years were excluded from the study.

### Data collection

The data was collected from June 3rd to June 28th, 2019. The study enlisted the participation of all surgical personnel who met the inclusion criteria. The study excluded 39 of the department’s 225 surgical professionals (surgeons, anesthetists, and theatre nurses); 28 were not physically accessible (on yearly or study leave), and seven consultants were no longer active in theatre. The 30-item questionnaire (S1 Questionnaire) was self-administered by 190 eligible surgical providers at KBTH. The majority of the providers took the survey at weekly departmental clinical meetings. The rest took them in the operating theaters, surgical wards, and the out-patients department. In all, providers were given privacy and were not allowed to discuss the survey while taking it. On average, providers completed the paper-based questionnaire in 15 minutes. The survey collected data on the providers’ socio-demographic factors (such as years of surgical experience, rank, and specialty), general information on checklist use (awareness, understanding of features, training, and use), and determinants of WHO SSC non-use. The questionnaire was based on Verwey and Gopalan’s [38] set of questions adapted from Fourcade et al.’s [13] previously identified barriers to using the SSC. These were assessed on a 5-point Likert scale (strongly agree to strongly disagree).

### Statistical analysis

The mean ± standard deviation (SD) for continuous variables and percentages for categorical variables were used to represent background characteristics, knowledge, and use of SSC. Where applicable, Pearson’s Chi-square and Fisher’s Exact Test were employed to look for differences between the groups. The variable frequency of usage was recategorized into a binary outcome variable, “always” and “not always,” to determine whether the checklist was always used. Answers to questions about barriers and perceptions were coded as “yes” (agree) or “no” (disagree). The “cannot tell” responses were combined with the “no” responses. Categorization into binary outcomes was done to facilitate the determination of crude prevalence ratios and the proportion of providers that always use the SSC. Two observations were excluded from the analysis because they had more than half of their data missing. The Chi-square test was utilized to find variables that were linked to the use of the SSC. Crude prevalence ratio (PR) estimates were calculated with a 95% confidence interval using log-binomial regression.

### Ethical considerations

The Institutional Review Board of Korle Bu Teaching Hospital granted ethical approval for the study (KBTH-IRB/00008/2019). The hospital administration was also contacted for permission. Informed consent was given verbally, and respondents were asked to sign a document indicating that they understood and agreed to all informed consent processes. This was done before enrolling in our research. All techniques followed the appropriate standards and regulations for conducting human population research.

## Results

### Background characteristics

A response rate of 97.9% was achieved, with 186 of the 190 questionnaires received being returned. Not every surgical provider answered every query. Table 1 lists the backgrounds and personal traits of the surgical professionals. Respondents were relatively evenly distributed across the different cadre of surgical professionals; surgeons (37%), anesthetists (36%), and theatre nurses (27%). Most providers (50%) were between the ages of 27 and 34. Surgeons had the highest percentage (16.2%) of responders between the ages of 45 and 64 years, while nurses had the lowest percentage (2.0%). A total of 97 respondents (52.7%) were men. The majority of anesthetists (59.1%) and nurses (80%) were female, but the majority of surgeons (88.2%) were male.

**Table 1:** Background characteristics of surgical professionals.

Variable	Type of surgical professional			
	Surgeon	Anesthetist	Nurse	Total
	N (%)	N (%)	N (%)	N (%)
**Respondents**	68(37)	66(35.9)	50(27.2)	184(100)
**Age**				
25–34	24(35.3)	33(50)	35(70)	92(50)
35–44	33(48.5)	25(37.9)	14(28)	72(39.1)
45–64	11(16.2)	8(12.1)	1(2)	20(10.9)
**Sex**				
Women	8(11.8)	39(59.1)	40(80)	87(47.3)
Men	60(88.2)	27(40.9)	10(20)	97(52.7)
**Educational status**				
Bachelor’s degree	31(45.6)	34(51.5)	23(46)	88(47.8)
Diploma	0(0)	0(0)	26(52)	26(14.1)
Postgraduate degree	37(54.4)	32(48.5)	1(2)	70(38)
**Surgical Unit**				
Neurosurgery	8(11.8)	0(0)	15(30)	23(12.5)
General Surgery	41(60.3)	0(0)	26(52)	67(36.4)
Urology	11(16.2)	0(0)	0(0)	11(6)
Pediatric Surgery	8(11.8)	0(0)	9(18)	17(9.2)
Anesthesia	0(0)	66(100)	0(0)	66(35.9)
**Rank of Medical Personnel**				
Consultant	20(29.4)	19(28.8)	0(0)	39(21.2)
General nurse	0(0)	0(0)	28(56)	28(15.2)
Perioperative nurse	0(0)	0(0)	22(44)	22(12)
Resident/Medical Officer	32(47.1)	34(51.5)	0(0)	66(35.9)
Specialist/Snr. Resident	16(23.5)	13(19.7)	0(0)	29(15.8)
**Years spent studying or working at the hospital (Mean±SD)**	7.2±5.8	7.6±6	6.4±4.2	7.1±5.5

The educational background of the surgical professionals varied, with most anesthetists having a bachelor’s degree (51.5%). In comparison, most surgeons (54.4%) had a postgraduate degree. However, among nurses, a diploma degree was the most frequently mentioned educational achievement (52%). Bachelor’s degree refers to a university degree in nursing or medicine; a postgraduate degree refers to any qualification obtained after a Bachelor’s degree, while a diploma refers to qualifications obtained from the Nursing Training College or equivalent institution other than the University. The general surgery unit employed around 36% of the responders, while the neurosurgery, pediatric surgery, and urology units employed about 13%, 9%, and 6%. Anesthetists working in the anesthesia division who were not affiliated with a particular surgical unit made up 35.9% of the workforce. Anesthetists comprised 51.5% of residents and medical officers, while 19.7% and 28.8% were specialists and consultants. Among surgeons, 47.1% were residents, 23.5% were specialists, and 29.4% were consultants. The average (SD) number of years spent working or studying at the hospital was 7.1 years (5.5), with anesthetists having the highest average of 7.6 years (6.0) and nurses having the lowest average of 6.4 years (4.2).

### Checklist utilization

All surgical professionals who were interviewed knew about and had ever used the WHO SSC. Nearly a third (30.4%) of surgical providers reported always utilizing the checklist for all surgical cases, as the WHO recommends. Compared to anesthetists (27.3%) and surgeons (26.5%), more nurses (40.0%) used the SSC always. On the other hand, surgeons made up around 43% of the staff who did not always use the SSC. About two-thirds (67.7%) of our respondents said that since the SSC’s implementation, they had not received any training on its use. The highest percentage of non-trained individuals were surgeons (80.9%), followed by anesthetists (66.7%) and nurses (50.0%). Self-reported knowledge of the checklist was highest among anesthetists (33.3%) than among nurses (32.0%) and surgeons (23.5%). The level of awareness, utilization, training, and expertise of surgical workers is shown in Table 2.

**Table 2:** Surgical provider reported utilization of the WHO SSC.

Variables	Surgical professional			
	Surgeon N (%)	Anesthetist N (%)	Nurse N (%)	Total N (%)
**Awareness of the WHO SSC**				
Yes	68(100)	66(100)	50(100)	184(100)
**Use of the WHO SSC**				
Yes	68(100)	66(100)	50(100)	184(100)
**How often WHO SSC is used**				
Always	18(26.5)	18(27.3)	20(40.0)	56(30.4)
Mostly	38(55.9)	39(59.1)	23(46.0)	100(54.3)
Sometimes	12(17.6)	9(13.6)	7(14.0)	28(15.2)
**Had training on the use of SSC**				
No	55(80.9)	44(66.7)	25(50)	124(67.4)
Yes	13(19.1)	22(33.3)	25(50)	60(32.6)
**Knowledge of SSC**				
Average	15(22.1)	17(25.8)	8(16)	40(21.7)
Good	37(54.4)	27(40.9)	26(52)	90(48.9)
Very good	16(23.5)	22(33.3)	16(32)	54(29.3)

### Beliefs associated with checklist utilization

Table 3 displays responses from surgical professionals to previously identified barriers to checklist use cross-tabbed against the surgical professionals’ category. The vast majority of surgical professionals felt the SSC was necessary (98.9%). A comparable number of respondents felt that surgical professionals needed to be trained in using the checklist (98.4%). Among respondents, the only barrier that showed a discernible difference across the various groups of surgical professionals was the perception that the SSC is challenging to integrate into staff members’ perioperative routines. Nurses were almost 16 times more likely than anesthetists and surgeons to report having trouble implementing the SSC into their perioperative routines (PR, 95% CI; 15.84, 2.13–117.82). The perceptions of the various groups of surgical staff about the problem of inadequate communication between anesthetists and surgeons also varied significantly. For example, anesthetists and nurses were 2.36 (95% CI; 1.04–5.34) and 2.86 (95% CI; 1.26–6.46) times more likely than surgeons to believe there was poor communication between anesthetists and surgeons.

**Table 3:** Cross-tabulation of identified barriers and perceptions against the type of surgical professional.

Responses	Yes n (%)	No n (%)	PR [95%CI]
**The SSC takes too long to complete**			
Surgeon	8(12.1)	58(87.9)	
Nurse	10(21.3)	37(78.7)	1.76 [0.75, 4.11]
Anesthesiologist	14(21.2)	52(78.8)	1.75 [0.79, 3.89]
**The SSC duplicates with other existing checks**			
Surgeon	14(26.4)	39(73.6)	
Nurse	11(36.7)	19(63.3)	1.39 [0.72, 2.66]
Anesthesiologist	9(20.5)	35(79.5)	0.77 [0.37, 1.62]
**There is poor communication between anesthetists and surgeon**			
Surgeon	7(10.9)	57(89.1)	
Nurse	15(31.3)	33(68.8)	2.86 [1.26, 6.46]
Anesthesiologist	16(25.8)	46(74.2)	2.36 [1.04, 5.34]
**It is unnecessary to use the SSC**			
Surgeon	1(1.5)	67(98.5)	
Nurse	0(0)	49(100)	
Anesthesiologist	1(1.5)	65(98.5)	1.03 [0.06, 16.13]
**The SSC is a waste of time**			
Surgeon	1(1.5)	67(98.5)	
Nurse	1(2)	48(98)	1.39 [0.09, 21.65]
Anesthesiologist	0(0)	66(100)	
**The SSC is difficult to incorporate into my perioperative routine**			
Surgeon	1(1.5)	65(98.5)	
Nurse	12(24)	38(76)	15.84 [2.13, 117.82]
Anesthesiologist	0(0)	66(100)	
**The SSC does not cover all risks e.g., skin preparation and postoperative pain prevention**			
Surgeon	54(88.5)	7(11.5)	
Nurse	36(75)	12(25)	0.85 [0.70, 1.02]
Anesthesiologist	54(87.1)	8(12.9)	0.98 [0.86, 1.12]
**It is difficult to find a coordinator for the checklist**			
Surgeon	20(30.8)	45(69.2)	
Nurse	16(34)	31(66)	1.11 [0.64, 1.90]
Anesthesiologist	18(29)	44(71)	0.94 [0.55, 1.61]
**I know whose responsibility it is to initiate the checklist**			
Surgeon	38(76)	12(24)	
Nurse	30(83.3)	6(16.7)	1.10 [0.89, 1.36]
Anesthesiologist	28(80)	7(20)	1.05 [0.84, 1.32]
**Staff needs to be trained in using the checklist**			
Surgeon	65(97)	2(3)	
Nurse	48(100)	0(0)	1.03 [0.99, 1.08]
Anesthesiologist	65(98.5)	1(1.5)	1.02 [0.96, 1.07]
**Team members’ attitude toward the questions on the checklist is not encouraging**			
Surgeon	30(52.6)	27(47.4)	
Nurse	25(51)	24(49)	0.97 [0.67, 1.40]
Anesthesiologist	34(56.7)	26(43.3)	1.08 [0.77, 1.50]
**Surgical personnel (surgeons) support the use of the checklist**			
Surgeon	57(93.4)	4(6.6)	
Nurse	45(93.8)	3(6.3)	1.00 [0.91, 1.11]
Anesthesiologist	37(74)	13(26)	0.79 [0.66, 0.95]
**Anesthetic personnel support the use of the checklist**			
Surgeon	61(93.8)	4(6.2)	
Nurse	42(87.5)	6(12.5)	0.93 [0.82, 1.06]
Anesthesiologist	65(98.5)	1(1.5)	1.05 [0.98, 1.12]
**Nursing personnel support the use of the checklist**			
Surgeon	63(95.5)	3(4.5)	
Nurse	48(96)	2(4)	1.01 [0.93, 1.09]
Anesthesiologist	59(96.7)	2(3.3)	1.01 [0.94, 1.09]
**Management supports the use of the checklist**			
Surgeon	29(87.9)	4(12.1)	
Nurse	44(95.7)	2(4.3)	1.09 [0.95, 1.25]
Anesthesiologist	27(93.1)	2(6.9)	1.06 [0.90, 1.24]

The results also demonstrate that the majority of respondents agreed with the claim that surgical professionals support the use of the checklist. Nurses (96.1%) and surgeons (87.6%) were rated as the most and least supportive of the SSC, respectively, among the categories of surgical professionals.

Table 4 displays the findings of factors that influence surgical professionals’ use of the checklist. In the bivariate analysis, utilization of the checklist was significantly associated with the following perceptions: it is difficult to find a coordinator for the checklist; there is poor communication between the anesthetist and surgeon; I know whose responsibility it is to initiate the checklist; team members believe that the SSC takes too long to complete; and the SSC does not cover all risks, such as skin preparation and postoperative pain. For instance, surgical professionals were less likely to consistently use the SSC if they felt that the checklist was too time-consuming to complete, that it did not account for all perioperative hazards, or that it was challenging to find a coordinator for the checklist. However, surgical professionals who concurred that they knew who was responsible for initiating the checklist were more inclined to use it in every circumstance. The use of checklists was also significantly associated with the personnel specialty unit and training on SSC use. Compared to employees at the Neurosurgery unit, those working in General Surgery, Urology, and Pediatric Surgery units were less likely to use the SSC constantly. Compared to employees who received no training, those who received SSC training were more likely to use the checklist consistently.

**Table 4:** Association between surgical provider’s beliefs or barriers and utilization of the WHO Surgical Safety Checklist.

		WHO SSC use		PR [95%CI]
		Yes	No	
The SSC takes too long to complete	Yes	3	29	0.27 [0.09, 0.81]
	No	51	96	
The SSC duplicates with other existing checks	Yes	8	26	0.66 [0.34, 1.29]
	No	33	60	
There is poor communication between anesthetist and surgeon	Yes	2	36	0.14 [0.04, 0.54]
	No	52	84	
It is unnecessary to use a SSC	Yes	2	0	3.42 [2.72, 4.28]
	No	53	128	
The SSC is a waste of time	Yes	0	2	
	No	55	126	
The SSC is difficult to incorporate into my perioperative routine	Yes	2	11	0.48 [0.13, 1.76]
	No	54	115	
The SSC does not cover all risks e.g., skin preparation and postoperative pain prevention	Yes	37	107	0.46 [0.30, 0.72]
	No	15	12	
It is difficult to find a coordinator for the checklist	Yes	5	49	0.22 [0.09, 0.52]
	No	51	69	
I know whose responsibility it is to initiate the checklist	Yes	40	56	2.60 [1.03, 6.59]
	No	4	21	
Staff needs to be trained in using the checklist	Yes	54	124	
	No	0	3	
Team members’ attitude toward the questions on the checklist is not encouraging	Yes	17	72	0.40 [0.24, 0.65]
	No	37	40	
Surgical personnel (surgeon) support the use of the checklist	Yes	45	94	2.16 [0.74, 6.30]
	No	3	17	
Anesthetic personnel support the use of the checklist	Yes	53	115	1.74 [0.49, 6.20]
	No	2	9	
Nursing personnel support the use of the checklist	Yes	53	117	0.73 [0.30, 1.76]
	No	3	4	
Management supports the use of the checklist	Yes	34	66	0.91 [0.36, 2.31]
	No	3	5	
Surgical Unit: Neurosurgery		15	8	
General Surgery		16	51	0.37 [0.22 0.62]
Urology		2	9	0.28 [0.08, 1.01]
Pediatric Surgery		5	12	0.45 [0.20, 1.00]
Anesthesia		18	48	0.42 [0.26, 0.69]
Training on using SSC	Yes	28	32	2.07 [1.35, 3.16]
	No	28	96	

## Discussion

Despite being well-documented as a high-impact safety measure, usage of the WHO SSC since its implementation continues to vary across different economic settings [32]. This study aimed to assess surgical professionals’ use of the WHO SSC and identify variables that contribute to its non-use so that recommendations may be tailored to promote its use in Ghana. Even though all respondents in our study were aware of the WHO SSC, only 30.4% said they always used it as intended. This finding is similar to one found in an Indian study, in which the majority of respondents praised the SSC’s positive effects (>96%) but observed a compliance rate of 40% among operating employees [39]. This self-reporting of low compliance to the WHO SSC supports the notion that even a massively positive attitude among employees does not imply compliance. According to Rogers and Shoemaker, the transmission of innovation, even when it has evident benefits, can be difficult and take a long time to spread [40]. As a result, the unique challenge for stakeholders has been to discover a mechanism to accelerate the rate of innovation diffusion. Other factors, other than recognition of the checklist’s merits, appear to affect its non-use in the operating room. Time-related concerns were revealed as a significant factor of checklist non-use in our study. Although nearly all employees (98.9%) agreed that using the SSC was necessary, about a fifth of them, especially anesthetists (21.2%) and nurses (20.8%) thought the checklist took too long to complete. Surgical professionals who believed the checklist took too long to complete were 80 percent less likely to use it all of the time. One of the primary barriers to checklist compliance found by Fourcade et al. [13] and reinforced by Kariyo et al. [41] in their research of African countries was the feeling that the checklist took too long to complete.

Although time was a significant determinant of SSC non-use among respondents, the majority of surgical staff (81.8%) did not believe the SSC takes too long to complete. It is worth noting the similarities between this discovery and that of Verwey & Gopalan [38], who acknowledged the difficulties in interpreting a comparable observation in their South African study. We reason that, while the SSC is designed to be quick, the extra few seconds spent outside of surgical personnel’s everyday routines at various stages during the procedure can be viewed as an “additional” task, which is sufficient to encourage non-use. The fact that substantially more nurses than surgeons and anesthetists acknowledged difficulty incorporating the SSC into their perioperative practice supports this conclusion. Nurses were about 16 times more likely than surgeons and anesthetists to have difficulty incorporating the SSC into their practice. According to the WHO SSC’s default design and suggested use, nurses have a greater role in completing the checklist, as they are responsible for reading out the items on the checklist and filling it out. This could explain why nurses had more difficulty incorporating the checklist into their daily routine than surgeons and anesthetists.

Most surgical professionals (84.3%) believe the SSC does not address all hazards, such as skin preparation and postoperative pain. In our study participants, this was a significant factor of non-utilization. Personnel who thought the SSC’s risk coverage was insufficient were 54% less likely to use the checklist. Furthermore, almost one-third of respondents (31.3%) said finding a coordinator for the checklist was difficult. Personnel who cited difficulty locating a coordinator were 78% less likely to use the SSC regularly than those who did not. These findings align with Fourcade et al.’s [13] study, which found that both impressions are essential. In their investigation, Verwey and Gopalan [38] did not find both characteristics as important impediments. When it comes to the obstacle of the checklist’s insufficient coverage, adjusting the content and application for varied situations is one area where intervention can help enhance compliance. Studies indicate that when the checklist is contextualized to the hospital’s operations, use improves [13]. In a multi-center study that looked at the interacting elements that influence WHO SSC use, it was discovered that a minor tweak to the checklist in the context of local hospital operating procedures could help enhance its use and make it easier to accept the checklist [42]. Few doctors in the study even advised creating a checklist that can be easily customized to match different surgical procedures to boost utilization. Furthermore, contextualizing a checklist to represent local operating room dynamics allows for consideration of aspects such as time, checklist integration into personnel routine, and duplication of existing procedures.

One of the WHO SSC’s primary goals is to improve communication among surgical team members [23]. In our study, anesthetists and nurses were 2.4 and 2.9 times more likely than surgeons to believe there was inadequate communication between anesthetists and surgeons, respectively. Additionally, the overwhelming majority of surgical professionals believed that the SSC was supported by nurses (96.1%), anesthetists (93.9%), and management (92.7%). In contrast, the perception of surgeons’ support for the use of the checklist had a lower majority (87.6%) and was significantly different among the groups of surgical professionals. These findings are in keeping with other studies and indicate an area for intervention [13, 39, 43], in contrast to Tostes & Galvao [44] who identified a lack of administrative and management support as one of the barriers to the use of the SSC. Educating surgical personnel on using the SSC offers a viable modality of intervention that can address communication issues and the disparate perspectives among surgical personnel [45].

Only a third (32.3%) of all respondents in our study had formal training in using the SSC. Among the various types of surgical workers, this was lowest among surgeons (19.8%), who were also the least supportive of the SSC. The low level of training on SSC use among personnel (32.3%) highlights an area of concern similar to that observed in a South African study among theatre staff [38]. Anwer et al.’s [46] review of surgical procedures in Pakistan also indicated a positive correlation between compliance and the training period, implying that training surgical professionals may be a crucial intervention to promote compliance. Interestingly, despite ongoing training, research in Ethiopia found that SSC compliance decreased by 65% over eight months following introduction [47]. This shows that training alone may not be sufficient to solve the problem of low compliance. Nearly all of our group (98.4%) believed that surgical staff needed to be trained, even though most (77.4%) said they knew the checklist either well or very well. It is possible that this finding indicates surgical professionals’ awareness of a problem with the sustainability and implementation of SSC use, which they believe can be addressed through training. Nonetheless, the results of our study appear to support a beneficial relationship between training and checklist use. The likelihood that surgical providers will always utilize the SSC was twice higher after training on checklist use (PR, 95% CI; 2.07, 1.35–3.16).

Additionally, we observed a strong relationship between surgical specialty and the use of the SSC. For example, professionals in the General Surgery and Pediatric Surgery units were 63% and 55% less likely to always use the checklist compared to the Neurosurgery unit. This is in line with research that evaluated how different subspecialties complied with the surgical checklist and was conducted across ten hospitals in Colorado [48]. The observed variation in SSC use among the specialties may further emphasize the need for context-specific checklist modifications and surgical professional-oriented interventions.

A few limitations may apply to our study. First is the use of convenience sampling in selecting our study population from a single public tertiary facility. Although this approach enhanced our total sample size, our findings may be center-specific and not generalizable to non-public or non-tertiary facilities. Second is the use of data from self-administered questionnaires, which were completed individually by the different categories of providers. This meant that we could not parse out the conflicting or reinforcing perceptions of checklist use amongst providers who work together as a team. Lastly, we cannot exclude social desirability bias in our study population and may have overestimated checklist awareness and utilization.

## Conclusion

Despite surgical personnel’s widespread knowledge of and familiarity with the WHO SSC, its intended use was inadequate, putting surgical patients at risk. The hurdles linked to the time required to complete the checklist, the inexhaustive coverage of risks, and the confusion in designating a checklist coordinator were the key drivers that caused the checklist’s non-use in our setting. Significant differences in perception between the two groups of surgical personnel suggest poor team communication, difficulty incorporating the SSC into a perioperative routine, and surgeons’ lack of support for using the SSC are areas of concern that could benefit from differential intervention. Overall, the determinants of non-use in our study environment are similar to those observed in other settings. As a result, there is a need to address socio-cultural hurdles unique to various settings that affect the checklist’s adoption and long-term viability. In this population, we advocate targeted and ongoing SSC training. Training should focus on adapting the checklist to the local environment and enhancing the overall performance of the various workers in the operation room as a team. With a focus on surgeons, training should also address communication, support, and role-playing deficiencies. Local coordinators should be recruited and assigned the task of championing the WHO SS checklist and regularly auditing and evaluating its use. This procedure will also reveal emergent concerns that can be addressed through more training or feedback.

## Supporting information

S1 Checklist World Health Organization’s Surgical Safety Checklist. (DOCX)

S1 Questionnaire Study questionnaire. (DOCX)

## References

[1] Nepogodiev D, Martin J, Biccard B, Makupe A, Bhangu A, Ademuyiwa A, et al. Global burden of postoperative death. The Lancet. 2019;393(10170):401. doi: 10.1016/S0140-6736(18)33139-8 30722955

[2] Weiser TG, Haynes AB, Molina G, Lipsitz SR, Esquivel MM, Uribe-Leitz T, et al. Size and distribution of the global volume of surgery in 2012. Bull World Health Organ. 2016 [accessed 2019 Oct 17];94:201–209. https://www.ncbi.nlm.nih.gov/pmc/articles/PMC4773932/pdf/BLT.15.159293.pdf. doi: 10.2471/BLT.15.159293 26966331 PMC4773932

[3] Pearse RM, Clavien PA, Demartines N, Fleisher LA, Grocott M, Haddow J, et al. Global patient outcomes after elective surgery: Prospective cohort study in 27 low-, middle- and high-income countries. British Journal of Anaesthesia. 2016;117(5):601–609. doi: 10.1093/bja/aew316 27799174 PMC5091334

[4] Biccard BM, Madiba TE, Kluyts HL, Munlemvo DM, Madzimbamuto FD, Basenero A, et al. Perioperative patient outcomes in the African Surgical Outcomes Study: a 7-day prospective observational cohort study. The Lancet. 2018;391(10130):1589–1598. doi: 10.1016/S0140-6736(18)30001-1 29306587

[5] World Alliance for Patient Safety. Safe surgery saves lives. 2008.

[6] Lancet T. WHO’s patient-safety checklist for surgery. The Lancet. 2008;372(9632):1. doi: 10.1016/S0140-6736(08)60964-2 18603137

[7] AbbottTEF, AhmadT, PhullMK, FowlerAJ, HewsonR, BiccardBM, et al. The surgical safety checklist and patient outcomes after surgery: a prospective observational cohort study, systematic review and meta-analysis. British Journal of Anaesthesia. 2018;120(1):146–155. doi: 10.1016/j.bja.2017.08.002 29397122

[8] Lilaonitkul M, Kwikiriza A, Ttendo S, Kiwanuka J, Munyarungero E, Walker IA, et al. Implementation of the WHO Surgical Safety Checklist and surgical swab and instrument counts at a regional referral hospital in Uganda—A quality improvement project. Anaesthesia. 2015;70(12):1345–1355. doi: 10.1111/anae.13226 26558855

[9] Vijayasekar C, Steele RJC. The World Health Organization’s surgical safety checklist. Surgeon. 2009;7(5):260–262. doi: 10.1016/s1479-666x(09)80001-2 19848057

[10] Vandijck D, Bergs J. The WHO surgical safety checklist: An innovative or an irrelevant tool? Acta Chirurgica Belgica. 2014;114(4):225–227. 26021415

[11] Mahajan RP. The WHO surgical checklist. Best Practice and Research: Clinical Anaesthesiology. 2011;25(2):161–168. doi: 10.1016/j.bpa.2011.02.002 21550541

[12] World Health Organization. WHO Guidelines for Safe Surgery 2009. Who. 2009:125. http://whqlibdoc.who.int/publications/2009/9789241598552_eng.pdf. doi: January 13, 2013 23762968

[13] Fourcade A, Blache JL, Grenier C, Bourgain JL, Minvielle E. Barriers to staff adoption of a surgical safety checklist. BMJ Quality and Safety. 2012. doi: 10.1136/bmjqs-2011-000094 22069112 PMC3285141

[14] Haynes AB, Weiser TG, Berry WR, Lipsitz SR, Breizat A-HS, Dellinger EP, et al. A Surgical Safety Checklist to Reduce Morbidity and Mortality in a Global Population. New England Journal of Medicine. 2009 [accessed 2018 Oct 15];360(5):491–499. https://www.nejm.org/doi/pdf/10.1056/NEJMsa0810119. 19144931 10.1056/NEJMsa0810119

[15] Naidoo M, Moodley J, Gathiram P, Sartorius B. The impact of a modified world health organization surgical safety checklist on maternal outcomes in a South African setting: A stratified cluster-randomised controlled trial. South African Medical Journal. 2017;107(3):248–257. doi: 10.7196/SAMJ.2017.v107i3.11320 28281432

[16] Weiser TG, Haynes AB, Dziekan G, Berry WR, Lipsitz SR, Gawande AA. Effect of A 19-Item Surgical Safety Checklist During Urgent Operations in A Global Patient Population. 2010 [accessed 2018 Oct 15]. www.annalsofsurgery.com. doi: 10.1097/SLA.0b013e3181d970e3 20395848

[17] Haynes AB, Edmondson L, Lipsitz SR, Molina G, Neville BA, Singer SJ, et al. Mortality Trends after a Voluntary Checklist-based Surgical Safety Collaborative. Annals of Surgery. 2017;266(6):923–929. doi: 10.1097/SLA.0000000000002249 29140848

[18] Bergs J, Hellings J, Cleemput I, Zurel Ö, de Troyer V, van Hiel M, et al. Systematic review and meta-analysis of the effect of the World Health Organization surgical safety checklist on postoperative complications. British Journal of Surgery. 2014 [accessed 2018 Sep 27];101(3):150–158. http://www.ncbi.nlm.nih.gov/pubmed/24469615. doi: 10.1002/bjs.9381 24469615

[19] Treadwell JR, Lucas S, Tsou AY. Surgical checklists: A systematic review of impacts and implementation. BMJ Quality and Safety. 2014;23(4):299–318. doi: 10.1136/bmjqs-2012-001797 23922403 PMC3963558

[20] Cassiana Gil Prates, Claudio Marcel Berdun Stadñik, Airton Bagatini, Rita Catalina Aquino Caregnato GMSS de M. Comparison of surgical infection rates after implementation of a safety checklist. Acta Paul Enferm. 2018;31(2):116–22. doi: 10.1046/j.1365-2087.1998.00089.x

[21] Molina G, Jiang W, Edmondson L, Gibbons L, Huang LC, Kiang M v, et al. Implementation of the Surgical Safety Checklist in South Carolina Hospitals Is Associated with Improvement in Perceived Perioperative Safety. Journal of the American College of Surgeons. 2016 [accessed 2018 Oct 13];222:725–736.e5. doi: 10.1016/j.jamcollsurg.2015.12.052 27049781

[22] Ramsay G, Haynes AB, Lipsitz SR, Solsky I, Leitch J, Gawande AA, et al. Reducing surgical mortality in Scotland by use of the WHO Surgical Safety Checklist. British Journal of Surgery. 2019. doi: 10.1002/bjs.11151 30993676

[23] Lingard L, Regehr G, Orser B, Reznick R, Baker GR, Doran D, et al. Evaluation of a preoperative checklist and team briefing among surgeons, nurses, and anesthesiologists to reduce failures in communication. Archives of surgery. 2008;143(1):12–17. doi: 10.1001/archsurg.2007.21 18209148

[24] Sewell M, Adebibe M, Jayakumar P, Jowett C, Kong K, Vemulapalli K, et al. Use of the WHO surgical safety checklist in trauma and orthopaedic patients. International Orthopaedics. 2011 [accessed 2018 Oct 15];35(6):897–901. https://www.ncbi.nlm.nih.gov/pmc/articles/PMC3103968/pdf/264_2010_Article_1112.pdf. doi: 10.1007/s00264-010-1112-7 20730425 PMC3103968

[25] Takala RSK, PAUNIAHO S, Kotkansalo A, Helmiö P, Blomgren K, Helminen M, et al. A pilot study of the implementation of WHO Surgical Checklist in F inland: improvements in activities and communication. Acta anaesthesiologica Scandinavica. 2011;55(10):1206–1214. doi: 10.1111/j.1399-6576.2011.02525.x 22092125

[26] Makary MA, Mukherjee A, Sexton JB, Syin D, Goodrich E, Hartmann E, et al. Operating room briefings and wrong-site surgery. Journal of the American College of Surgeons. 2007;204(2):236–243. doi: 10.1016/j.jamcollsurg.2006.10.018 17254927

[27] Pugel AE, Simianu V v, Flum DR, Patchen Dellinger E. Use of the surgical safety checklist to improve communication and reduce complications. Journal of Infection and Public Health. 2015 [accessed 2018 Oct 13];8:219–225. doi: 10.1016/j.jiph.2015.01.001 25731674 PMC4417373

[28] de Vries EN, Prins HA, Crolla RMPH, den Outer AJ, van Andel G, van Helden SH, et al. Effect of a Comprehensive Surgical Safety System on Patient Outcomes. New England Journal of Medicine. 2010 [accessed 2018 Oct 15];363(20):1928–1937. https://www.nejm.org/doi/pdf/10.1056/NEJMsa0911535. 21067384 10.1056/NEJMsa0911535

[29] Sivathasan N, Rakowski KRM, Robertson BFM, Vijayarajan L. The World Health Organization’s ‘Surgical Safety Checklist’: should evidence-based initiatives be enforced in hospital policy? JRSM Short Reports. 2010 [accessed 2018 Oct 15];1(5):1–5. www.drfosterhealth.co.uk. doi: 10.1258/shorts.2010.010007 21103132 PMC2984370

[30] Urbach DR, Govindarajan A, Saskin R, Wilton AS, Baxter NN. Introduction of Surgical Safety Checklists in Ontario, Canada. New England Journal of Medicine. 2014;370(11):1029–1038. doi: 10.1056/NEJMsa1308261 24620866

[31] Lübbeke A, Hovaguimian F, Wickboldt N, Barea C, Clergue F, Hoffmeyer P, et al. Effectiveness of the Surgical Safety Checklist in a High Standard Care Environment. Medical Care. 2013 [accessed 2018 Sep 30];51(5):425–429. http://content.wkhealth.com/linkback/openurl?sid=WKPTLP:landingpage&an=00005650-201305000-00008. doi: 10.1097/MLR.0b013e31828d1489 23579353

[32] Borchard A, Schwappach DLB, Barbir A, Bezzola P. SYSTEMATIC REVIEW AND META ANALYSIS A Systematic Review of the Effectiveness, Compliance, and Critical Factors for Implementation of Safety Checklists in Surgery. 2012 [accessed 2018 Nov 18]. http://links.lww.com/SLA/A57. doi: 10.1097/SLA.0b013e3182682f27 22968074

[33] Vats A, Vincent CA, Nagpal K, Davies RW, Darzi A, Moorthy K. Practical challenges of introducing WHO surgical checklist: UK pilot experience. BMJ (Clinical research ed.). 2010 [accessed 2018 Sep 30];340:b5433. http://www.ncbi.nlm.nih.gov/pubmed/20071413. doi: 10.1136/bmj.b5433 20071413

[34] Russ SJ, Sevdalis N, Moorthy K, Mayer EK, Rout S, Caris J, et al. A Qualitative Evaluation of the Barriers and Facilitators Toward Implementation of the WHO Surgical Safety Checklist Across Hospitals in England Lessons From the &quot;Surgical Checklist Implementation Project&quot; 2014 [accessed 2018 Oct 20]. www.nrls.npsa.nhs.uk. doi: 10.1097/SLA.0000000000000793 25072435

[35] Melekie TB, Getahun GM. Compliance with Surgical Safety Checklist completion in the operating room of University of Gondar Hospital, Northwest Ethiopia. BMC Research Notes. 2015;(1):1–7. doi: 10.1186/s13104-015-1338-y 26285824 PMC4544783

[36] Matei A, Mouhajer M, Tontoh H, Ampah N, Quartson EM, George RB, et al. Implementation of the WHO surgical safety checklist in a west african teaching hospital: A quality improvement initiative. Update in Anaesthesia. 2020;35:5–10.

[37] Korle Bu Teaching Hospital. 2016 Annual report. 2017. http://ebrary.ifpri.org/cdm/ref/collection/p15738coll2/id/131159. doi: 10.2499/9780896292628

[38] Verwey S, Gopalan PD. An investigation of barriers to the use of the world health organization surgical safety checklist in theatres. South African Medical Journal. 2018;108(4):336–341. doi: 10.7196/SAMJ.2017.v108i4.12780 29629686

[39] Aggarwal N, Dhaliwal N, Joshi B. To evaluate the use of surgical safety checklist in a tertiary referral obstetrics center of Northern India. Obstetrics & Gynecology International Journal. 2018 [accessed 2019 Jun 29];9(2). http://medcraveonline.com/OGIJ/OGIJ-09-00318.php. doi: 10.15406/ogij.2018.09.00318

[40] Rogers EM, Shoemaker F. Diffusion of innovation: a cross-cultural approach. 1983. http://www.experience-capitalization.net/bitstream/handle/123456789/83/diffusion-of-innovations.pdf?sequence=1&isAllowed=y. doi: 10.1007/s10661-014-3885-4

[41] Kariyo PC, Hightower J, Bosco J, Ii N, Mwikisa C. Challenges facing the introduction of the WHO surgical safety checklist: A short experience in African countries. Africa Health Monitor. 2013;(March).

[42] Gagliardi AR, Straus SE, Shojania KG, Urbach DR. Multiple Interacting Factors Influence Adherence, and Outcomes Associated with Surgical Safety Checklists: A Qualitative Study Courvoisier DS, editor. PLoS ONE. 2014 [accessed 2019 Jun 29];9(9):e108585. https://dx.plos.org/10.1371/journal.pone.0108585. doi: 10.1371/journal.pone.0108585 25260030 PMC4178177

[43] Papaconstantinou HT, Jo C, Reznik SI, Smythe WR, Wehbe-Janek H. Implementation of a surgical safety checklist: impact on surgical team perspectives. Ochsner Journal. 2013;13(3):299–309. 24052757 PMC3776503

[44] Tostes MF do P, Galvao CM. Surgical safety checklist: benefits, facilitators, and barriers in the nurses’ perspective. Lista de verificacao de seguranca cirurgica: beneficios, facilitadores e barreiras na perspectiva da enfermagem. 2019;40(spe):e20180180. 10.1590/1983-1447.2019.20180180 30652803

[45] Haynes AB, Weiser TG, Berry WR, Lipsitz SR, Breizat AHS, Dellinger EP, et al. Changes in safety attitude and relationship to decreased postoperative morbidity and mortality following implementation of a checklist-based surgical safety intervention. BMJ Quality and Safety. 2011;20(1):102–107. doi: 10.1136/bmjqs.2009.040022 21228082

[46] Anwer M, Manzoor S, Muneer N, Qureshi S. Compliance and effectiveness of WHO surgical safety check list: A JPMC audit. Pakistan journal of medical sciences. 2016;32(4):831. doi: 10.12669/pjms.324.9884 27648023 PMC5017086

[47] Bashford T, Reshamwalla S, McAuley J, Allen NH, McNatt Z, Gebremedhen YD. Implementation of the WHO surgical safety checklist in an Ethiopian referral hospital. Patient safety in surgery. 2014;8(1):1–12. 24678854 10.1186/1754-9493-8-16 PMC4022152

[48] Biffl WL, Gallagher AW, Pieracci FM, Berumen C. Suboptimal compliance with surgical safety checklists in Colorado: A prospective observational study reveals differences between surgical specialties. Patient safety in surgery. 2015;9(1):5. doi: 10.1186/s13037-014-0056-z 25642287 PMC4312457

