# Supplementary material for: Surgical provider-reported reasons for utilization of the World Health Organization’s Surgical Safety Checklist at a tertiary hospital in Ghana
Source: PLOS Glob Public Health. 2023 Jan 12;3(1):e0001143. doi: 10.1371/journal.pgph.0001143 (PMC10021622; doi:10.1371/journal.pgph.0001143)
Supplement: S1 Checklist — (DOCX) [file pgph.0001143.s001.docx]

# S1 Checklist – WHO SURGICAL SAFETY CHECKLIST


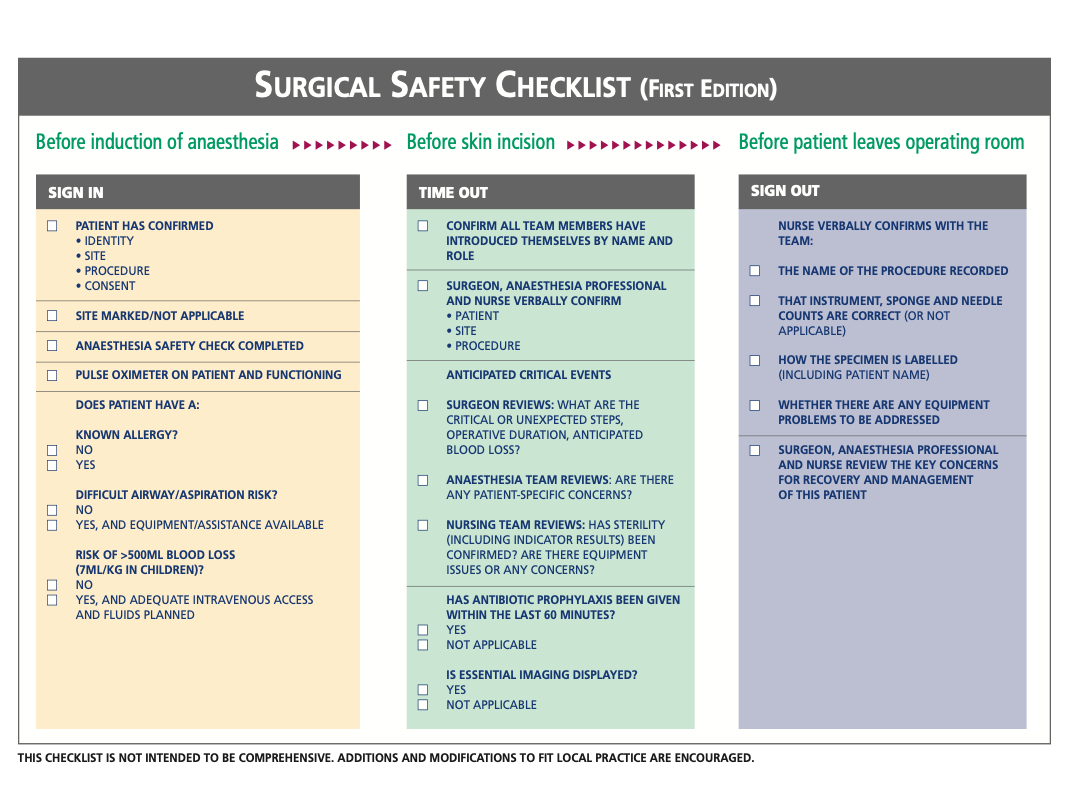


Source: ﻿WHO Patient Safety | WHO Guidelines for Safe Surgery 2009, Page 98
