# Supplementary material for: Surgical provider-reported reasons for utilization of the World Health Organization’s Surgical Safety Checklist at a tertiary hospital in Ghana
Source: PLOS Glob Public Health. 2023 Jan 12;3(1):e0001143. doi: 10.1371/journal.pgph.0001143 (PMC10021622; doi:10.1371/journal.pgph.0001143)
Supplement: S1 Questionnaire — (DOCX) [file pgph.0001143.s002.docx]

**STUDY QUESTIONNAIRE**

**Title: *The World Health Organization’s Surgical Safety Checklist: Acceptability Among Surgical Personnel in Korle Bu Teaching Hospital in Accra.***

I kindly ask you to answer the following questions and statements regarding the use of the WHO Surgical Safety Checklist. By doing so you contribute to a better scientific understanding of the topic above and improvement in surgical safety. Information provided will be kept strictly confidential. Thank you.

**Date of interview: ………………………………………………..**

| **SECTION A: DEMOGRAPHIC CHARACTERISTICS** | | | | | | | | | |
| --- | --- | --- | --- | --- | --- | --- | --- | --- | --- |
|  | Age in years | …………………… | | | | | | | |
|  | Sex | 01. Male ( ) 02. Female ( ) | | | | | | | |
|  | Religion | 01.Christianity ( ) 02. Islamic ( )  03. Traditional ( )  04. Other (please specify) ........................................................... | | | | | | | |
|  | Educational status | 01. Diploma ( )  02. Bachelor’s degree ( )  03. Post-graduate degree ( ) | | | | | | | |
|  | Surgical Unit | 01. General Surgery ( )  02. Urology ( )  03. Neurosurgery ( )  04. Paediatric Surgery ( )  05. Anaesthesia ( )  Other (please specify) ……………………………………... | | | | | | | |
|  | Surgical team (if applicable for surgeons) | Please state: …………………………… | | | | | | | |
|  | Type of Surgical Personnel | 1. Surgeon ( ) 2. Anaesthesiologist ( ) 3. Nurse ( ) | | | | | | | |
|  | Level of specialization | 1. Consultant ( ) 2. Specialist/Snr. Resident ( ) 3. Resident/Medical Officer ( ) 4. Peri-operative nurse ( ) 5. General nurse ( ) 6. Student nurse ( ) | | | | | | | |
|  | Years spent in studying or working at the hospital | ……………………………………….. | | | | | | | |
|  | Satisfied with current profession | 01. Very satisfied ( ) 02. Satisfied ( ) 03. Neither ( )  04.Unsatisfied ( ) 05. Very unsatisfied ( ) | | | | | | | |
| **B. GENERAL INFORMATION ON THE WHO SSC** | | | | | | | | | |
|  | Are you aware of the WHO SSC? | | 01. Yes ( ) 02. No ( ) | | | | | | |
|  | Do you use the WHO SSC? | | 01. Yes ( ) 02. No ( ) | | | | | | |
|  | If YES, how often do you use the WHO SSC? | | 01. Always ( ) 02. Mostly ( )  03. Sometimes ( ) 04. Rarely ( ) | | | | | | |
|  | Have you had training on the use of the SSC? | | 01. Yes ( ) 02. No ( ) | | | | | | |
|  | How would you describe your knowledge of the SSC? | | 01. Very good ( ) 02. Good ( )  03. Average ( ) 04. Bad ( )  05. Very bad ( ) | | | | | | |
| **C. BARRIERS TO THE USE OF WHO SSC** | | | | | | | | | |
| The under listed factors does not allow you to effectively use the SSC (kindly tick) | | | | | | | | | |
|  | | | **Strongly agree** | **Agree** | **Can’t tell** | | **Disagree** | | **Strongly disagree** |
|  | The SSC takes too long to complete | |  |  |  | |  | |  |
|  | The SSC duplicates with other existing checks | |  |  |  | |  | |  |
|  | There is poor communication between anaesthetist and surgeon | |  |  |  | |  | |  |
|  | It is unnecessary to use a SSC | |  |  |  | |  | |  |
|  | The SSC is a waste of time | |  |  |  | |  | |  |
|  |  | | **Strongly agree** | **Agree** | **Can’t tell** | | **Disagree** | | **Strongly disagree** |
|  | The SSC is difficult to incorporate into my perioperative routine | |  |  |  | |  | |  |
|  | The SSC does not cover all risks e.g. skin preparation and postoperative pain prevention | |  |  |  | |  | |  |
|  | It is difficult to find a co-ordinator for the checklist | |  |  |  | |  | |  |
|  | I know whose responsibility it is to initiate the checklist | |  |  |  |  | |  | |
|  | Staff needs to be trained in using the checklist | |  |  |  |  | |  | |
|  | Team members attitude towards the questions on the checklist is not encouraging | |  |  |  |  | |  | |
|  | Surgical personnel (surgeon) support the use of the checklist | |  |  |  |  | |  | |
|  | Anaesthetic personnel support the use of the checklist | |  |  |  |  | |  | |
|  | Nursing personnel support the use of the checklist | |  |  |  |  | |  | |
|  | Management support the use of the checklist | |  |  |  |  | |  | |

**THANK YOU**
